# MAC Protocol for Ad Hoc Networks Using a Genetic Algorithm

**DOI:** 10.1155/2014/670190

**Published:** 2014-07-21

**Authors:** Omar Elizarraras, Marco Panduro, Aldo L. Méndez, Alberto Reyna

**Affiliations:** Universidad Autónoma de Tamaulipas, UAMRR, Carr. Reynosa-San Fernando S/N, Colonia Arcoiris, 88779 Reynosa, TAMPS, Mexico

## Abstract

The problem of obtaining the transmission rate in an ad hoc network consists in adjusting the power of each node to ensure the signal to interference ratio (SIR) and the energy required to transmit from one node to another is obtained at the same time. Therefore, an optimal transmission rate for each node in a medium access control (MAC) protocol based on CSMA-CDMA (carrier sense multiple access-code division multiple access) for ad hoc networks can be obtained using evolutionary optimization. This work proposes a genetic algorithm for the transmission rate election considering a perfect power control, and our proposition achieves improvement of 10% compared with the scheme that handles the handshaking phase to adjust the transmission rate. Furthermore, this paper proposes a genetic algorithm that solves the problem of power combining, interference, data rate, and energy ensuring the signal to interference ratio in an ad hoc network. The result of the proposed genetic algorithm has a better performance (15%) compared to the CSMA-CDMA protocol without optimizing. Therefore, we show by simulation the effectiveness of the proposed protocol in terms of the throughput.

## 1. Introduction

In a wireless ad hoc network, the nodes can communicate with each other without the support of infrastructure. Since the wireless channel is shared by all the nodes in the network, a medium access control (MAC) plays an important role in coordinating access among the nodes so that information gets through from one node to another [1]. Usually each node is able to communicate with each other's node when all nodes are spread around a geographic range. However, nodes could spread over larger geographic range than the communication signal can reach. In this case, the nodes could have communication over multiple hops. However, there is only one medium that is shared by all the nodes that are in the same radio communication range and the radio frequency bandwidth is limited. Furthermore, packet collisions are unavoidable due to the fact that traffic arrivals are random and there is nonzero propagation time between transmitters and receivers. Therefore, MAC schemes are used to coordinate the access to the channel in the network [2].

The tendency of the MAC protocols for wireless ad hoc networks is using adaptive systems to adjust the transmission parameters (multirate) and the objective is to maximize the throughput in the use of the channel. Rate adaptation is indispensable to optimally exploit the scarce wireless resources under instable channel conditions. Rate adaptation consists of assessing the wireless channel conditions and selecting the most appropriate data rate. Moreover, in the MAC protocols, the low throughput in the region of low traffic is because there is no more information for sending (this is not due to errors of the multiuser interference). So the system performance is limited by the access technique used in wireless ad hoc networks [3]. To address this problem, we use a rate adaptation to optimize throughput requirements. Then, the problem can be formulated as an optimization problem; that is, minimize the resources consumption considering the power, interference, data rate, and energy ensuring the signal to interference ratio in an ad hoc network. The multirate in ad hoc networks has been addressed in [4–9]; these protocols were proposed to maximize the throughput by adapting the rate based on the channel, but they do not address the energy issue.

The MAC protocol plays a critical role in a wireless ad hoc network considering bandwidth efficiency, resolving collisions, resources allocation, power transmission, interference, energy, data rate, and distance. In most standardized wireless ad hoc networks, such as in the widely deployed IEEE 802.11 networks, only one node is allowed to transmit in a given time slot, while in CSMA-CDMA (carrier sense multiple access-code division multiple access) protocol more than one node can transmit; that is, more than one simultaneous transmission can be achieved. Hence, CSMA-CDMA MAC protocol is employed for multihop wireless ad hoc networks [10–13].

Furthermore, power control is applied in a wireless ad hoc network to control transmission range (distance between the source and destination); on the other hand, it is useful to keep low interference levels to the neighboring nodes. Because CDMA systems are interference limited, power control also serves as a tool for interference management in CDMA systems to guarantee success of multiple concurrent transmissions [1]. Another parameter closely related with power transmission is the energy consumption. In critical environments such as military or rescue operations, where ad hoc networks will be typically used, conserving of battery power will be vital in order to make the network operational for long durations. Recharging or replacing batteries will not often be possible. The majority of work in the literature focuses on the protocol design and performance evaluation in terms of traditional metrics such as throughput, delay, power, energy, and distance. In this case, the literature analyzes these terms by separate. Some protocols related to these routing metrics have been proposed in [14–18].

In order to improve the performance (throughput) of an ad hoc network, we use a MAC protocol based on CDMA with an efficient evolutionary algorithm for transmission rate election. In this evolutionary optimization algorithm, we control the transmission rate and handle the spreading factor or processing gain (*P*_*G*_) for a MAC scheme based on CSMA-CDMA ad hoc networks and we take a perfect control power into account.

Modern evolutionary optimization techniques have aroused great interest among the scientific and technical community in a wide variety of fields for the last years because of their ability to solve problems with a nonlinear and nonconvex dependence of design parameters. Several optimization techniques have emerged in the past two decades that mimic biological evolution or the way biological entities communicate in nature. Some of these algorithms have been used successfully in many electromagnetism and network problems with many constraints and nonlinear processes. The most representative algorithm includes genetic algorithms (GA) [19], among others.

Therefore, in this work, we present a genetic algorithm that solves the problem of power combining, interference, data rate, and energy ensuring the signal to interference ratio in an ad hoc network and we use Dijkstra's algorithm to make the search and discovery of the route.

The remainder of this paper is organized as follows. In Section 2, we introduce the model for the MAC protocol based on CSMA-CDMA considering a perfect power control. The operation of proposed protocol is presented in Section 3.  Section 4 presents the genetic algorithm for the transmission rate election.  Section 5 presents the simulations performed and the results obtained. Finally, we present the conclusions of our work in Section 6.

## 2. MAC Protocol Considering a Perfect Power Control

In a system based on CSMA-CDMA, the CSMA protocol is used to access the channel and reserve it for a communication between two nodes. Instead, the function of CDMA is to assign a code to each node so they can transmit simultaneously.

The CSMA protocol operation is as follows: when a node wants to transmit its data, it monitors the channel state. If the channel is idle for a time period that is equal to a distributed interframes space, the node transmits the request-to-send (RTS) packet after a random period called backoff. If the channel is not idle, the node defers its transmission. If the destination node detects an RTS packet, it responds with a clear-to-send (CTS) packet. The transmission will only start after the CTS packet is correctly received. Moreover, the packets RTS and CTS carry the information of the duration of the packet to be transmitted. This information can be read by each node, which is then able to update their network allocation vector (NAV) containing the information of the period of time in which the channel will remain busy.

In the CDMA protocol, the simultaneous transmission of packet by different nodes is allowed. Therefore, it is necessary for a code assignment mechanism to assign distinct codes to different nodes. Several code assignment mechanisms have been previously proposed [20, 21] and we have used the receiver-transmitter-based mechanism.

Considering the previously mentioned, in this paper, the MAC protocol for ad hoc networks is based on CSMA-CDMA with different transmission rates. Furthermore, we consider a perfect control power and an error-free channel. The operation of the protocol MAC is as follows.

Assuming that node A has generated a packet and it needs to transmit to node B, then

1. node A contends for the allocation of a code. This code is selected randomly from a code table;
2. node A sends a RTS message informing the neighboring nodes of its intention to transmit, code selected, transmission duration, and destination node (node B). Node A changes its state to receive the response message (CTS) from node B. If node A does not receive the CTS message, it assumes that there was a collision and applies the backoff mechanism. This step (Step 2) will run until it has received the reply from node B or until the lifetime of the packet has expired;
3. node B receives the RTS message; it sends the reply message (CTS) and informs the neighboring nodes that will be occupied (attending node A);
4. node A determines the number of active neighboring nodes. With this number, it can obtain an optimal transmission rate according to the genetic algorithm. Then, it sends data packet to node B;
5. once B receives the packets, it sends an ACK (acknowledgement) message to inform node A that the packets were successfully received and the code is released;
6. if A does not receive the ACK message, it returns to Step 2 until the transmission turns successful or until the packet expires.

Furthermore, we need to propose an algorithm to obtain the transmission rate for each node. Therefore, if a node needs to transmit a packet, it calculates the number of neighboring nodes trying to transmit in a time slot. With this number, it is possible to obtain the best transmission rate combination for each node [3]. This combination of transmission rates should maximize the throughput and it is based on the nodes that transmit at different rates as well as the bit error probability (*P*_*b*_) or bit error rate (BER) as it is often called. The use of the Gaussian approximation to determine *P*_*b*_ is based on the argument that the decision statistic and the multiple access interference may be modeled as a Gaussian random variable. Therefore, considering that the system is limited by interference and noise is neglected, the bit error probability used for a fixed rate of transmission is given by [22]

(1) $$\begin{array}{c} P_{b}\left(n\right)=Q\left(\sqrt{3\frac{P_{G}}{n-1}}\right), \end{array}$$

where *n* is the number of simultaneous nodes, *P*_*G*_ is the processing gain and it is defined as the ratio of the transmission bandwidth to the information bandwidth, and *Q* is the error function given by

(2) $$\begin{array}{c} Q\left(x\right)=\frac{1}{\sqrt{2\pi }}\overset{\infty }{\underset{x}{\int }}e^{-u^{2}/2}du, \end{array}$$

where (2) is in terms of *u*, and this indicates the variable of the function to be integrated and defined in [23].

The correctly detecting probability of a packet containing *L* bits is

(3) $$\begin{array}{c} P_{d}\left(n\right)={\left[1-P_{b}\left(n\right)\right]}^{L}. \end{array}$$

In the CSMA-CDMA protocol, when the channel has a low traffic, a high processing gain is not necessary in the system because there is a low multiuser interference. Then, the processing gain (protection against multiuser interference) depends on the traffic conditions in the channel. Therefore, the system performance can be improved by using an adaptive transmission rate according to load conditions in the channel. Therefore, through genetic algorithms, it is possible to obtain the rate at which each node must transmit in an ad hoc network compared to [3] where it used exhaustive search.

Up to now, we have analyzed the CSMA-CDMA protocol with perfect power control to obtain the transmission rate for each node. In next section, we present the problem of power combining, interference, data rate, and energy ensuring the signal to interference ratio in a multihop ad hoc wireless network for the MAC protocol based on CSMA-CDMA as medium access technique with the ability to route packets.

## 3. Proposed Protocol

### 3.1. Operation

Assuming that node A has generated a packet and it needs to transmit to node B, which is out of node A transmission range, then

1. node A contends for the allocation of a code. This code is selected randomly from a code table. If a primary collision is present, then the nodes use a backoff mechanism to retransmit [24];
2. node A performs the route discovery based on the minimum distance, the same way as the link state routing protocol (Dijkstra). This means that the node source finds the route to its destination hop by hop through the minimum path until it reaches its destination node;
3. once the route is obtained, the algorithm optimizes the power, interference, transmission rate, and energy of the nodes that forward packets to destination node B;
4. node A sends the RTS message to the next node of the route. This process is repeated consecutively until it reaches node B. Node A changes its state to receive the CTS message;
5. node B receives the RTS message and it responds with a CTS message;
6. node A receives the CTS message and starts sending data packets. If node A receives no response from node B, it assumes that there was collision and retransmits the RTS message;
7. once the RTS-CTS dialog is completed, B starts to receive data packets and responds with an ACK message to inform node A that the packets have been received successfully;
8. if A receives no acknowledgement (ACK) from B, it waits for a random time to retransmit and repeat the process from Step 4;
9. once B receives the packets, it sends an ACK message to inform node A that the packets were successfully received and if there are no more packets to send, the code is released.

### 3.2. System Model

It is considered a wireless ad hoc network where there are *M* nodes participating in routing and *N* active nodes in the system. It should be mentioned that *M* nodes that participate in routing are determined in the phase of path discovery, and each node knows the neighbor nodes. This path discovery is assumed to be executed at the beginning of the simulation. We considered a link state routing algorithm, and all nodes know the detailed information of the network, topology, nodes neighboring, distance between them, and so forth. Moreover, the gain on the communication link between node *i* and node *j* is given by *g*_*ij*_. All the *g*_*ij*_ are positive. The transmission powers of node *i* and node *j* are denoted by *p*_*i*_ and *p*_*j*_, respectively. The current signal to interference ratio (SIR) [25] between node *i* and node *j*, *γ*_*ij*_, is the ratio between the power received from transmitter *i* at receiver *j* and the received interference caused by neighboring nodes plus noise power at receiver *j*, and it is determined by

(4) $$\begin{array}{c} \gamma_{ij}=\frac{W}{R_{ij}}\frac{g_{ij}p_{ij}}{\sum_{x=1,x\neq j,x\neq i}^{N}g_{jx}p_{jx}+\sigma_{j}},\;i=1,2,3,\ldots ,M, \end{array}$$

where *R*_*ij*_ is the data rate used to transmit from node *i* to node *j*, *W* is the total spread spectrum bandwidth occupied by CDMA, *σ*_*j*_ is the noise power at receiver *j*, and *p*_*ij*_ and *p*_*jx*_ are, respectively, the received powers of the transmissions between nodes *i* and *j* and nodes *j* and *x*.

On the other hand, if noise power at receiver *j* is neglected because its value is very small, then we have

(5) $$\begin{array}{c} \gamma_{ij}=\frac{W}{R_{ij}}\frac{g_{ij}p_{ij}}{\sum_{x=1,x\neq j,x\neq i}^{N}g_{jx}p_{jx}},\;i=1,2,3,\ldots ,M, \end{array}$$

and the channel gain is determined by

(6) $$\begin{array}{c} g_{ij}=d_{ij}^{-\propto }, \end{array}$$

where *d*_*ij*_ is the distance between nodes *i* and *j* and *α* is the attenuation coefficient.

We assume that each node should achieve the target SIR, *γ*_*ij*_^*t*^, from node *i* along the route to node *j* as follows:

(7) $$\begin{array}{c} \gamma_{ij}\geq \gamma_{ij}^{t},\;i=1,2,\ldots ,M,\;\;i\neq j. \end{array}$$

A power vector **P** and a transmission rate vector **R** are used such that they satisfy the criterion expressed in (7), where the power vector must comply as follows:

(8) $$\begin{array}{c} p_{\min}\leq p_{i}\leq p_{\max}. \end{array}$$

The **R** vector must satisfy the next criterion as follows:

(9) $$\begin{array}{c} R_{\min}\leq R_{ij}\leq R_{\max}. \end{array}$$

Considering that each node can choose its rate transmission and power within *K* possibilities, one has

(10) $$\begin{array}{c} p_{i}\in \left\{p_{i}^{1},p_{i}^{2},\ldots ,p_{i}^{K}\right\},\\ R_{i}\in \left\{r_{i}^{1},r_{i}^{2},\ldots ,r_{i}^{K}\right\}. \end{array}$$

## 4. Genetic Algorithm

**Figure 1:**
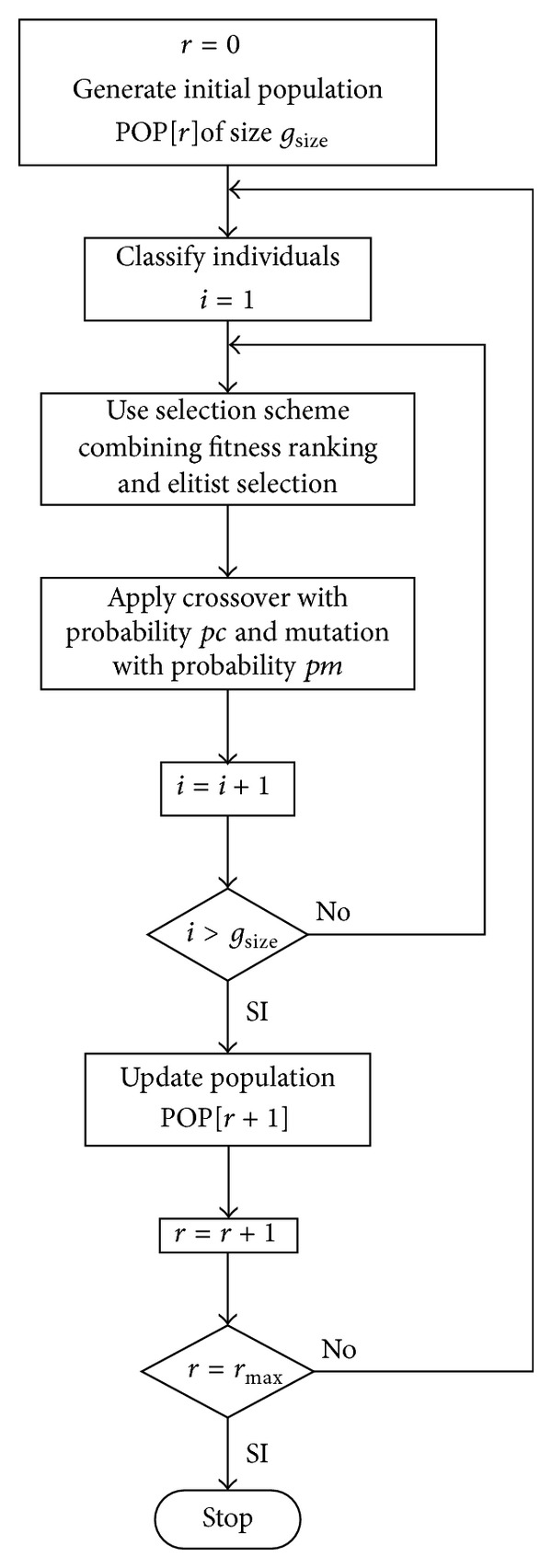
Flow chart for the evolutionary optimization algorithm.

The main purpose of this study is to solve the problem of power combining, interference, data rate, and energy ensuring the signal to interference ratio in an ad hoc network. For this purpose, we propose to use a population-based stochastic procedure denominated genetic algorithm (GA) [19]. We chose this algorithm for its easiness of implementation. The procedure for the used GA technique (Figure 1) is described as follows.

The function Generate Initial Population randomly and uniformly generates a set of individuals.

The main idea in Classify Individuals is to rank the individuals according to their fitness value.

A selection scheme combining fitness ranking and elitist selection [19] is implemented instead of a common weighted roulette wheel selection.

The function Update Population assigns ranks to individuals in the population generated by the union of parents and children. This is in order to hold the best individuals in each generation. Golberg [19] explains the procedures involved in each step of this algorithm in detail. The individual representations as well as the crossover and mutation operators are explained in the following subsections.

### 4.1. Individual Representation and Decoding

#### 4.1.1. Perfect Power Control

For a number of desired transmission rate combinations, *t*, with a population *P* of size *n*, each individual (combination of transmission rates) is expressed as

(11) P=[C1C2⋮Cn],

where *C*_1_ ⋯ *C*_*n*_ represents each individual as potential solution. Moreover, *C* represents the combinations of transmission rates as individuals. Consider

(12) $$\begin{array}{c} C=\left[k_{1R}\cdots k_{tR}\right], \end{array}$$

where *k* nodes transmit at *t* times the basic rate (*R*).

On the other hand, the bandwidth, *W*, is given as

(13) $$\begin{array}{c} W=P_{G}\cdot R, \end{array}$$

where *P*_*G*_ is processing gain, *R* is the transmission rate, and the bandwidth (*W*) must be constant.

So the basic transmission rate is expressed in terms of processing gain as follows:

(14) $$\begin{array}{c} R=\frac{W}{P_{G}}. \end{array}$$

Therefore, the individuals in (11) are encoded in binary form so that the individual of population (12) is transformed into a single binary string.

#### 4.1.2. Power Combining

In this case, we consider the transmission rates and powers as individuals and potential solutions to the previously presented problem. Furthermore, they must comply with target SIR (7).

The vector **R** and vector **P** are transformed into binary numbers or strings; that is, they are encoded in a binary form. The chromosomes *A*_*i*_ and *B*_*i*_ are used to represent the vectors **R** and **P**, respectively, where *i* represents an individual. Moreover, *A*_*i*_ = *a*_1_, *a*_2_,…, *a*_*l*_, where *a*_1_, *a*_2_,…, *a*_*l*_ is the binary representation of length *l* for the rate transmission *R*_*i*_, and *B*_*i*_ = *b*_1_, *b*_2_,…, *b*_*l*_, where *b*_1_, *b*_2_,…, *b*_*l*_ is the binary representation for power *p*_*i*_ [25].

The individuals for power population and rate transmission population are given by

(15) A=[A1A2⋮AT],B=[B1B2⋮BT],

where *T* is the population size.

### 4.2. Genetic Operators

The used genetic operators are standard; the well-known two-point crossover [19] along with a single mutation where a locus is randomly selected and the allele is replaced by a random number is uniformly distributed in the feasible region.

### 4.3. Objective Function

#### 4.3.1. Perfect Power Control

The objective function (of) for maximizing the performance of the transmission rates combination is given by

(16) $$\begin{array}{c} \text{of}=\max S. \end{array}$$

Evaluating the performance *S* (throughput) for a transmission rates combination *t*, considering a perfect power control, is determined as the average of the correctly detecting probability of each node that transmits with different rates [22] as follows:

(17) $$\begin{array}{c} S_{t}=\overset{t_{\max}}{\underset{t}{\sum }}n_{tR}P_{d,tR}L, \end{array}$$

where *S*_*t*_ is the performance for the given combination of transmission rates, *n*_*tR*_ is number of nodes transmitting *t* times the transmission basic rate *R*, *P*_*d*,*tR*_ is the correctly detecting probability of a packet transmitted at *t* times the transmission basic rate *R*, *L* is packet length, and *t*_max⁡_ is the maximum value that can increase the basic rate.

Therefore, the procedure to find the optimal transmission rate is as follows.

1. Generate a population of random combinations of transmission rates.
2. Calculate the fitness (*S*_*t*_) of the population generated.
3. Apply genetic operators to the population by keeping the fittest individuals (best solutions).
4. Iterate Steps 2 and 3 the number of generations desired.

The result of this algorithm for each number of nodes is stored in a table. Therefore, depending on the number of the nodes that are transmitting in an instant of time, the nodes automatically will select the appropriate transmission rate.

#### 4.3.2. Power Combining

The objective function (of) used to minimize the resources consumption in each hop is as follows:

(18) $$\begin{array}{c} \text{of}=\min\left(\text{abs}\left(\gamma_{ij}-\gamma_{ij}^{t}\right)+E_{ij}\right). \end{array}$$

To evaluate each individual (power and rate) and the energy consumed through a path, we apply (19) (fitness). This equation represents an evaluation function for each possible solution obtained from the transmission rate and power calculated by each node. It is important to mention that, through this formulation, the genetic algorithm will tend to choose those solutions that require lower power, lower energy, and higher transmission rate, depending on the quality of the link (SIR). Consider

(19) $$\begin{array}{c} {\text{Fit}}_{i}=\text{abs}\left(\gamma_{ij}-\gamma_{ij}^{t}\right)+E_{ij}, \end{array}$$

where *E*_*ij*_ is the energy needed to transmit from node *i* to node *j*. To calculate the energy, we consider that the power transmission between nodes *i* and *j* is *p*_*ij*_, *R*_*ij*_ is the transmission rate used to transmit from node *i* to node *j*, *P*_*d*_(*γ*_*ij*_) is the probability of correctly detecting a packet, with *γ*_*ij*_ being equal to the SIR of link (*i*, *j*), and *L* denotes the packet length. Moreover, the number of transmissions necessary to receive a packet correctly is a random variable, *D*. If all transmissions are statistically independent, *D* is a geometric random variable. So the expected value of *D* is *E*[*D*] = 1/*P*_*d*_(*γ*_*ij*_). The total transmission time required for correct reception is the random variable *DL*/*R*_*ij*_. With the transmitted power *p*_*ij*_, the energy expended is the random variable *p*_*ij*_ *DL*/*R*_*ij*_ with expected value *E*[*D*]*Lp*_*ij*_/*R*_*ij*_ = *Lp*_*ij*_/[*R*_*ij*_ *P*_*d*_(*γ*_*ij*_)]. Therefore, energy *E*_*ij*_ is obtained by [26]

(20) $$\begin{array}{c} E_{ij}=\frac{LP_{ij}}{R_{ij}P_{d}\left(\gamma_{ij}\right)}. \end{array}$$

Energy (20) is in function of the transmission rate, and *P*_*d*_(*γ*_*ij*_) is given by

(21) $$\begin{array}{c} P_{c}\left(\gamma_{ij}\right)={\left(1-2{\text{BER}}_{ij}\right)}^{L}, \end{array}$$

where BER_*ij*_ is the bit error rate for the link (*i*, *j*). The BER is expressed as follows:

(22) $$\begin{array}{c} {\text{BER}}_{ij}={0.5e}^{-\gamma_{ij}/2}. \end{array}$$

Therefore, the main steps of the procedure of the proposed optimization algorithm are as follows.

1. Generate populations of transmission rate (**R**) and power (**P**).
2. Calculate the fitness of the population.
3. Manipulate the individuals by genetic operators keeping the elite individuals.
4. Repeat Steps 2 and 3 until the desired number of generations has been reached.

## 5. Simulations and Results

The first simulation is considering a perfect power control and the process of simulation was achieved by a program in MATLAB, on a personal computer, which considers a single hop ad hoc wireless network. We consider 160 nodes that are placed randomly in a square area of side length 150 meters. In this simulation, the nodes generate packets using a Pareto distribution. Additionally, we consider that the nodes are fixed. The preview concepts are applied to the simulation. Furthermore, a chip rate of 4.096 Mcps, four transmission rates (16, 32, 64, and 128 kbps), four processing gains (256, 128, 64, and 32), and the basic transmission rate of 16 kbps are employed.

**Figure 2:**
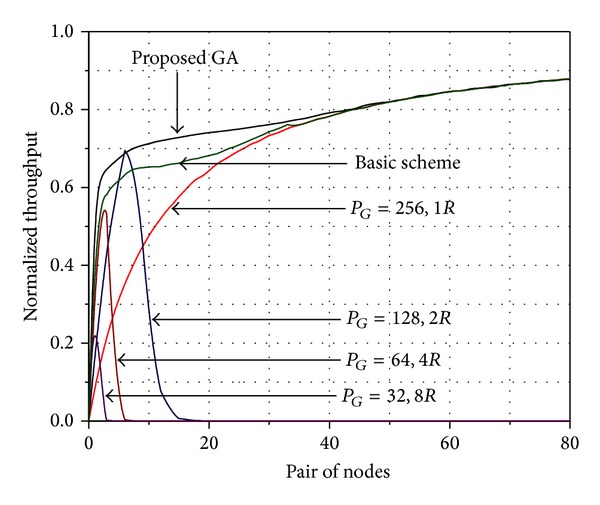
Throughput of the CSMA-CDMA protocol considering perfect power control.

For the genetic algorithm implemented, a population size of 50 individuals is used with 50 generations. Furthermore, crossover and mutation rates of 0.9 and 0.05 were used, respectively. These parameters have been selected following the recommendations of De Jong [27] and Grefenstette [28]. Furthermore, elitism is implemented in the genetic algorithm (GA) to ensure that the fitness of the population will not diminish from one generation to another. Elitism guarantees that the best individuals from the current generation are going to survive to the next generation. Therefore, with elitism is united the population of parents and children and half of them selected. The result of the simulation is shown in Figure 2.

Figure 2 shows the throughput behavior with proposed genetic algorithm (GA) for transmission rate election, and we observe a better performance with respect to fixed rates and the throughput is normalized with respect to maximum capacity of the system. Furthermore, Figure 2 illustrates that our proposed GA achieves improvement of 10% as compared with the basic scheme [13]. This is because the transmission rate with the basic scheme is obtained in the handshaking phase, where the node attempts to transmit with the maximum value of rate and if there is a fail in the handshaking phase, then the node decreases its transmission rate. When the node has success in the handshaking phase, it increases its transmission rate. Therefore, the basic scheme is a trial-and-error process. Unlike the basic scheme, in the proposed GA, the optimum transmission rate of each node is obtained and ensures that it will not affect the multiuser interference. Furthermore, during low traffic channel, nodes increase their transmission rates. As traffic increases, processing gain also increases, obtaining as a result a dynamic CDMA system bandwidth control.

The following simulation considers the combination of power, interference, data rate, and energy. The maximum power is 100 mW and the minimum power is 5 mW. The thermal noise is neglected. The target SIR (signal to interference ratio) was set to 3.918 dB. The attenuation coefficient *α* was set to 2. The channel gain only is in function of the distance. Therefore, fading is not considered. The transmission rates are 1200, 1800, 2400, 4800, 7200, 9600, and 14400 bps. The network was considered as a multihop network for this simulation, in which the routing technique used for routing data packets is based on minimum path. In this work, Dijkstra's algorithm for routing is implemented.

The genetic algorithm assigns powers and transmission rates considering the standard IS-95. The population size is of 50 individuals; crossover probability and mutation are of 0.95 and 0.05, respectively [27, 28]. The termination criterion is 50 generations. The individual length for the power was set to 7 bits and in the case of transmission rate is 3 bits.

**Figure 3:**
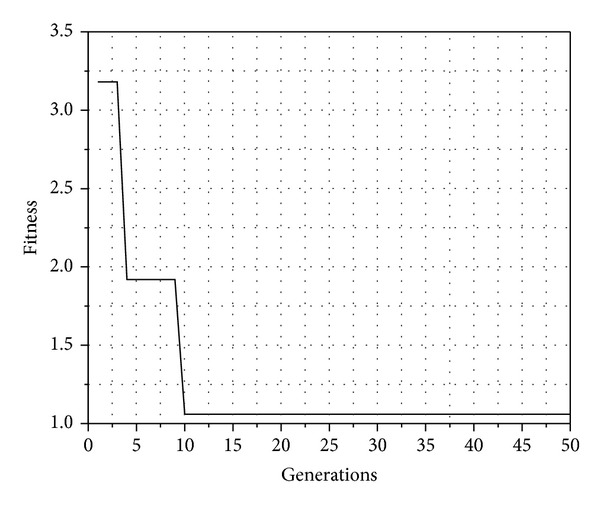
Fitness optimization.

An obtained result of the genetic algorithm is shown in Figure 3. This result confirms the effectiveness of the proposed algorithm.

**Figure 4:**
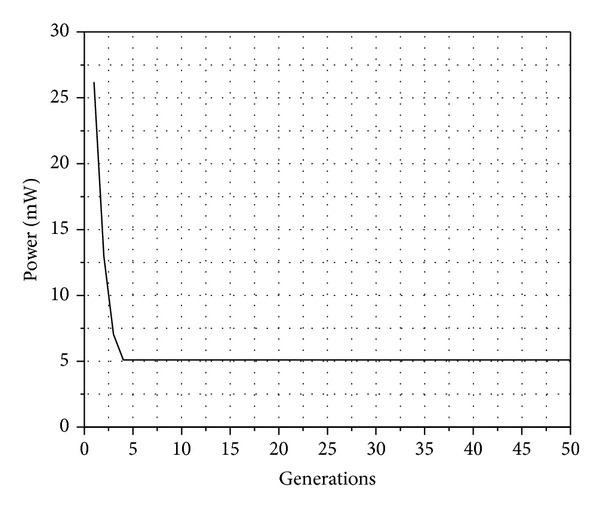
Behavior of the average power.

Figure 3 shows that when fitness decreases SIR approaches to the desired value; therefore, the node only uses the resource needed to reach the destination node without interfering with their neighbors. This is shown in Figure 4.

**Figure 5:**
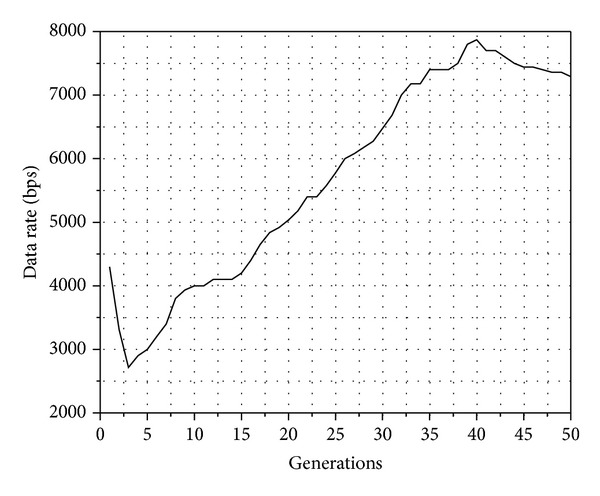
Behavior of transmission rate.

Figure 4 illustrates the behavior of power with respect to generation number using genetic algorithms. It is observed how the power is minimized and thus the node does not cause interference to neighboring nodes or expend more energy than necessary. Furthermore, another consideration of the genetic algorithm is to increase the transmission rate, and this behavior can be seen in Figure 5. The behavior shown in Figure 5 is the result of the genetic algorithm through 50 generations. In this graphic, we can see that the data rate is increased, while the power is decreased (Figure 4), which means that the SIR is fitted to a target with optimum parameters to achieve the best performance and throughput by the MAC protocol.

**Figure 6:**
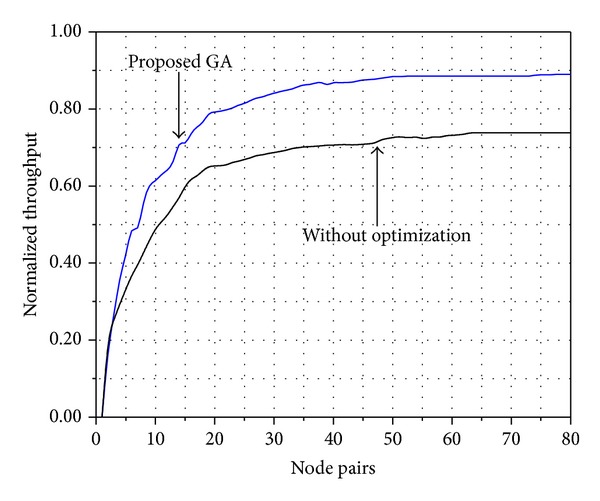
Throughput of CDMA ad hoc network using genetic algorithm (GA).

Furthermore, Figure 6 shows the throughput of proposed protocol where codes are used to grant the channel access and genetic algorithms are applied to guarantee the signal to interference ratio (SIR) for a successful transmission. Figure 6 shows the throughput behavior with proposed genetic algorithm (GA) for transmission multirate. The throughput is normalized with respect to maximum capacity of the system. Furthermore, during low traffic channel, nodes increase their transmission rates. As traffic increases, processing gain also increases, obtaining as a result a dynamic CDMA system bandwidth control. The proposed algorithm is compared with the CSMA-CDMA protocol without optimization. In CSMA-CDMA without optimization, as in [29], the nodes perform the RTS/CTS process and they adjust the power to reach their neighboring nodes but the SIR and the minimum energy are not guaranteed. Therefore, the CSMA-CDMA without optimization has a lower performance compared to our proposed GA (15%).

## 6. Conclusions

This paper has presented a MAC protocol based on CSMA-CDMA considering a perfect power control. A genetic algorithm is used to obtain the optimal transmission rate for each node in an ad hoc network. In this proposed protocol, we control the transmission rate according to the offered traffic. The simulation results demonstrate that proposed MAC protocol performs better than traditional CSMA-CDMA with fixed rates and the proposed protocol outperforms conventional protocols for single hop. Moreover, a shortest path routing protocol is applied in this paper when the nodes communicate with each other using multihop links. The method of genetic algorithms is used to optimize the network resource. On the other hand, the paper presents an efficient MAC protocol based on CSMA-CDMA to achieve high performance.
